# Endotheliopathy is associated with higher levels of cell-free DNA following major trauma: A prospective observational study

**DOI:** 10.1371/journal.pone.0189870

**Published:** 2017-12-19

**Authors:** David N. Naumann, Jon Hazeldine, Robert J. Dinsdale, Jon R. Bishop, Mark J. Midwinter, Paul Harrison, Sam D. Hutchings, Janet M. Lord

**Affiliations:** 1 Academic Department of Military Surgery and Trauma, Royal Centre for Defence Medicine, Queen Elizabeth Hospital, Birmingham, United Kingdom; 2 NIHR Surgical Reconstruction and Microbiology Research Centre, Queen Elizabeth Hospital, Birmingham, United Kingdom; 3 Institute of Inflammation and Ageing, University of Birmingham, Birmingham, United Kingdom; 4 Department of Surgery, University of Queensland, Rural Clinical School, Bundaberg, Queensland, Australia; 5 Department of Intensive Care Medicine, Kings College Hospital, Denmark Hill, London, United Kingdom; Klinikum rechts der Isar der Technischen Universitat Munchen, GERMANY

## Abstract

**Background:**

Cell free deoxyribonucleic acid (cfDNA) has been proposed as a biomarker of secondary complications following trauma. Raised thrombomodulin and syndecan-1 levels have been used to indicate endotheliopathy, and are associated with inflammation, coagulopathy, and mortality. The current study aimed to analyse the association between cfDNA and biomarkers of endotheliopathy in a cohort of trauma patients, and whether raised levels of cfDNA were associated with poorer clinical outcomes.

**Methods:**

Serum thrombomodulin and syndecan-1 were used as biomarkers of endotheliopathy and compared to plasma cfDNA in trauma patients from two prospective longitudinal observational studies. Cohort A (n = 105) had a predicted injury severity score (ISS) >8, and had blood sampled within 1h of injury and at 4–12h. Cohort B (n = 17) had evidence of haemorrhagic shock, and had blood sampled at a median time of 3.5h after injury. Relationships between biomarkers were tested using multivariable linear regression models that included the covariates of gender, age, ISS, Glasgow Coma Scale, lactate, systolic blood pressure, and heart rate. A model was fitted to investigate whether changes in cfDNA were associated with similar changes in endothelial biomarkers.

**Results:**

The mean age was 41 (SD 19), and the median ISS was 25 (IQR 12–34). There was a significant association between cfDNA levels and both syndecan-1 and thrombomodulin levels (both *p*<0.001). This was independent of all covariates except for ISS, which significantly correlated with cfDNA levels. 50 ng/ml change in syndecan-1 and 1 ng/ml change in thrombomodulin corresponded to 15% and 20% increases in cfDNA levels respectively (both *p*<0.001). Patients who died had significantly higher prehospital and in-hospital cfDNA levels (both *p*<0.05).

**Conclusions:**

Raised cfDNA levels are associated with markers of endotheliopathy following trauma, and are associated with mortality. This relationship is present within the first hour of injury, and a change in one biomarker level is reflected by similar changes in the others. These findings are in keeping with the hypothesis that circulating DNA and endothelial injury share a common pathway following trauma.

## Introduction

Cell-free deoxyribonucleic acid (cfDNA) is defined as extracellular DNA present within the circulation. It consists of a combination of nuclear and mitochondrial DNA (mtDNA) that enters the circulation following cell necrosis [1], tissue injury, and the generation of extracellular traps by activated immune cells such as neutrophils [2] and monocytes [3]. cfDNA may be elevated due to bacterial infection, and mtDNA shares structural similarities with pathogen–associated molecular patterns (such as those derived from bacteria). These fragments may therefore activate the innate immune response following aseptic injury, and contribute in part to the systemic inflammatory response observed following trauma [4–7]. The presence of cfDNA may also give rise to coagulopathy by coagulation factor activation, platelet aggregation, and inhibition of fibrinolysis [8, 9], whilst neutrophil extracellular traps (NETs) may cause further tissue injury and endothelial barrier dysfunction [10]. cfDNA has received interest as a biomarker following injury in relation to mortality [1, 11], organ failure [12], inflammation [2], and sepsis [13].

Syndecan-1 and thrombomodulin have been reported as biomarkers of endothelial cell and glycocalyx injury in multiple recent studies of trauma [14–16]. The former is a transmembrane heparan sulfate proteoglycan that forms part of the endothelial glycocalyx, and the latter is an integral membrane protein present on the surface of endothelial cells throughout the circulation. Raised levels of these biomarkers are associated with coagulopathy [14, 15, 17, 18], inflammation [15], poor microcirculatory flow[19], organ failure[20], and mortality [15–17].

Experimental models have demonstrated that mtDNA directly increases endothelial permeability through both neutrophil-independent and dependent pathways [21], and that it may induce a pro-thrombotic phenotype within the endothelium [22]. These findings suggest that there is likely to be a relationship between cfDNA and endotheliopathy, and that each may be mutually detrimental to the other. Indeed there is some preclinical evidence that endothelial cell damage by cfDNA is dose-dependent [23]. Other investigators have reported an association between endotheliopathy and elevation of cfDNA levels on admission to hospital following injury [15, 24, 25], but to our knowledge this has not been investigated within the pre-hospital period following trauma.

The current study aimed to investigate the levels of cfDNA and biomarkers of endotheliopathy following injury in a prospective cohort of trauma patients, and whether these biomarkers were associated with each other over time, from the pre-hospital period to hospital admission. We also aimed to investigate whether there was an association between levels of cfDNA and clinical outcomes, and with markers of coagulopathy.

We hypothesised that higher levels of biomarkers of endotheliopathy would be associated with higher levels of cfDNA, that this would be true even within the first hour of injury, and that there would be an association with markers of coagulopathy. We further hypothesised that an increase or decrease over time in each biomarker would be associated with the same directional changes in the others, and that raised cfDNA levels would be associated with poorer clinical outcomes. We hypothesised that patients who received a blood transfusion or had surgery would have increased levels of cfDNA when compared to those who did not.

## Methods

### Study design and setting

The current study combines data from two prospective longitudinal observational studies of trauma patients conducted at a single Major Trauma Centre site in the UK (University Hospitals Birmingham NHS Foundation Trust, Birmingham); these included patients from the Brain Biomarkers after Trauma (BBATS)[20] (REC reference: 13/WA/0399) and MICROSHOCK[19, 26] (REC reference: 14/YH/0078) studies. The former study (Cohort A) included patients with a predicted ISS >8, regardless of mechanism of injury or blood product requirement between May 2014 and February 2017. The latter (Cohort B) included patients with traumatic haemorrhagic shock requiring blood product transfusion, with a lactate > 2 mmol/l, and a requirement for intensive care between July 2015 and January 2017.

### Capacity and consent

As all patients lacked capacity to consent for study participation at the time of study enrolment, subjects were enrolled under the guidance of the Mental Health Act 2005 and the Declaration of Helsinki. Both the MICROSHOCK and BBATS studies were approved to gain consent to participate from a physician in charge of the care of the patient, but unrelated to the studies (Professional Consultee). Where appropriate, agreement for study participation was also obtained from a close friend or family of the patient (Personal Consultee). Ultimately, if the patient regained capacity, they were asked for consent to remain within the study, and for their previous data to be retained. If they did not regain capacity, then previous permissions from their Professional or Personal Consultee remained extant.

### Study time points

**Fig 1:**
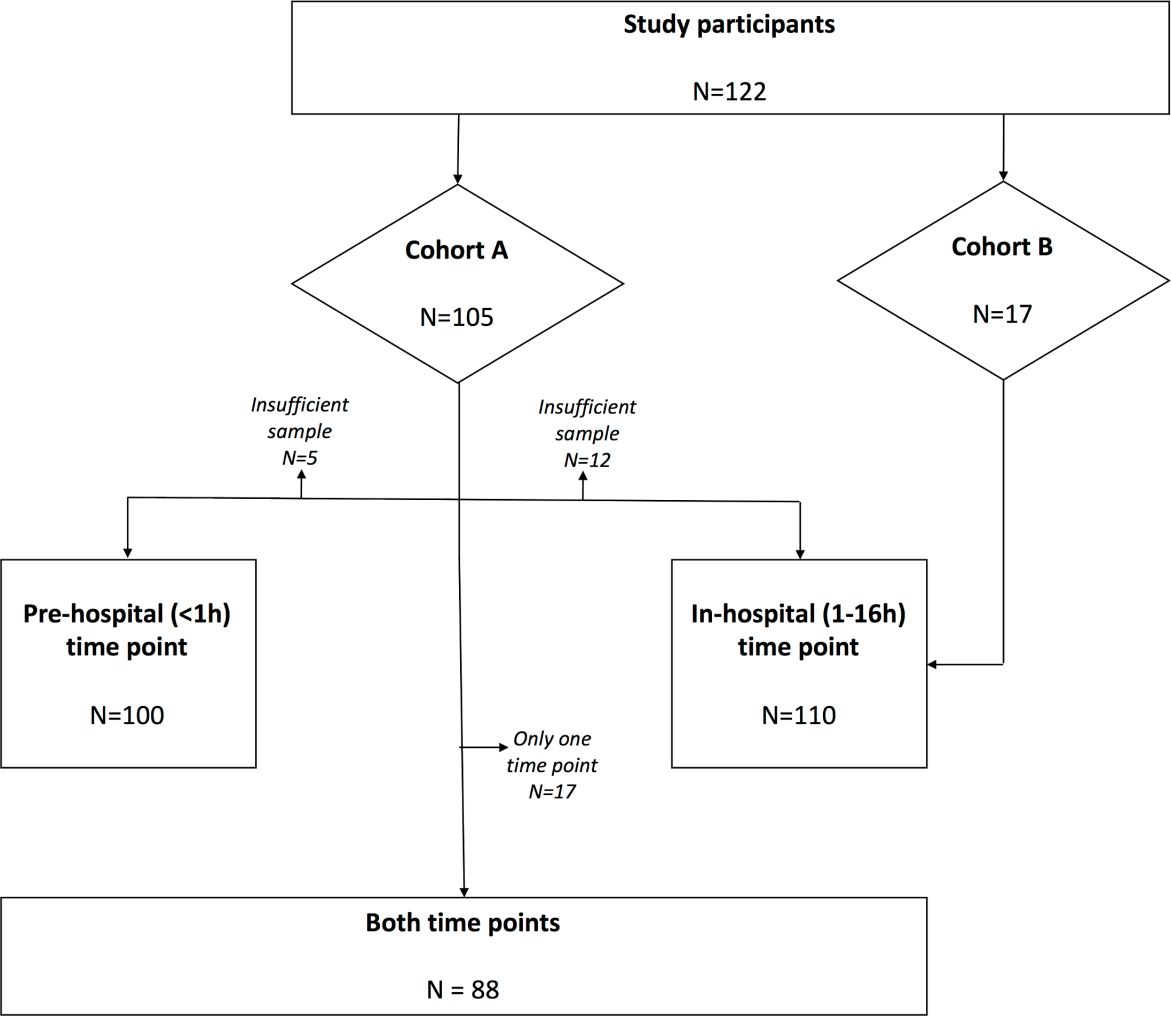
Flow diagram of study patients and samples available for analysis.

Patients in Cohort A all had blood sampled within the first 1 hour of injury by pre-hospital personnel (median time 44 (range 14–60) minutes). They then had a further sample taken between 4–12 hours following injury. Patients in Cohort B had blood sampled as soon as possible following admission to hospital, with a median time to sample of 3.5 (range 1–16) hours. For the purposes of the current study, two time points were investigated, as illustrated in Fig 1: (i) Pre-hospital (< 1 hour) (Cohort A only); and (ii) the first day of admission to hospital (1–16h) (Cohorts A and B).

### Blood sampling and preparation

All patients underwent venepuncture to obtain blood samples for research purposes using BD Vacutainers^®^ (Becton Dickinson, Oxford, UK). For serum and plasma preparation, blood was collected into vacutainers containing z-serum clotting activator or a 1/10 volume of 3.2% trisodium citrate respectively. After 30 minutes at room temperature, serum samples were centrifuged for 10 minutes at 1,620 x g. Plasma samples were centrifuged twice in order to minimise platelet contamination. First, samples were centrifuged for 20 minutes at 2,000 x g, then the top two thirds of fluid were spun again for 2 minutes at 13,000 x g. All centrifugation was performed at 4°C. Aliquots of serum and plasma were stored at -80°C, until they were analysed *en masse*.

### Measurement of endothelial biomarkers

The concentrations of thrombomodulin (CD 141) and syndecan-1 (CD 138) in serum samples were quantified using commercially available enzyme-linked immunosorbent assay kits (Abcam, Cambridge, MA) in precise accordance with manufacturer instructions. Each 96 well plate included a standard curve to interpolate the concentrations of biomarkers, as well as blank and control wells to validate the results.

### Measurement of cell-free DNA

In order to measure the cfDNA concentration in plasma samples, a fluorometric assay using SYTOX® Green Dye (Life Technologies, Cheshire, UK) was used as described previously [13]. For each plate, a standard curve was generated using samples of λ-DNA (Fisher Scientific, UK) ranging from 0–1000 ng/ml. To ensure that the assay was reliable between samples and assays, the inter-assay and intra-assay coefficients of variation were calculated; these were 5.3% and 5.1% respectively. 140 μl of SYTOX® Green Dye (Final concentration 1 μM) was added to 10 μl of plasma, and incubated for 10 minutes at room temperature in the dark. All samples were run in duplicate. Fluorescence was then measured using a BioTek Synergy 2 fluorometric plate reader (NorthStar Scientific Ltd, UK). Calibration was set at 485 nm and 528 nm for excitation and emission respectively. The average values of the duplicate wells were then used to derive cfDNA concentrations by interpolating from the standard curve.

### Data collection

Demographic and clinical data were obtained prospectively for all patients using a combination of electronic medical records and bedside medical notes. Data included age, sex, comorbidities, body mass index (BMI), smoking and alcohol status, injury severity score (ISS), mechanism of injury, lactate, systolic blood pressure, heart rate (HR), international normalised ratio (INR), partial thromboplastin time (PTT) ratio, and Glasgow Coma Scale (GCS). A Charlson Comorbidity Index was calculated for all patients based on the presence of comorbidities. Since it is possible that blood transfusion might also increase levels of cfDNA in transfused patients due to the cfDNA within donated blood products[27], patients who had been transfused between time points were recorded. Similarly, since surgery may also increase levels of cfDNA due to direct injury, patients who had surgery between time points were noted.

### Outcomes

Outcomes included 30-day mortality, thromboembolic events, as well as hospital-free and ICU-free days (calculated as 30 minus the number of days in hospital and ICU respectively).

### Data analysis

Normality of data was tested using the Shapiro-Wilk test. Continuous data are presented as mean and standard deviation (SD) for normal data, and median and interquartile range (IQR) for non-normal data. Categorical data are presented as N (%). Continuous data are compared using *t*-tests for normal data and Mann-Whitney U tests for non-normal data as appropriate. Categorical data are compared using Fisher’s exact test. Correlations have been tested using Spearman’s rank correlation co-efficient. The relationships between cfDNA and endothelial biomarkers were examined at both time points using multivariable linear regression models that included the covariates of gender, age, ISS, GCS, lactate, SBP, and HR. In order to determine whether levels of endothelial biomarkers followed the same directional change as cfDNA between time points, a model was fitted to examine the value of cfDNA at the second time point as a function of the change in Syndecan-1 and thrombomodulin values, modified by other covariates (gender, age, ISS, GCS, lactate, SBP, and HR) and the value of cfDNA at the first time point. A *p*-value of <0.05 was considered significant. Analyses were performed using R version 3.2.2 (http://www.r-project.org) and GraphPad Prism version 7.0 (GraphPad Software, California, USA).

## Results

### Patient characteristics

There were 122 patients included (105 from Cohort A and 17 from Cohort B), with a mean age of 41 (SD 19) and median ISS of 25 (IQR 12–34). A flow diagram of patients and available cfDNA at each time point is illustrated in Fig 1. Five of the patients in Cohort A did not have sufficient plasma and serum samples at the prehospital time point for comparison between biomarkers, making the denominator for the prehospital time point N = 100. There were a further 12 patients in Cohort A that had insufficient samples for the second time point, making the denominator for the in-hospital time point N = 110 (N = 93 from Cohort A, and N = 17 from Cohort B). There were therefore 88 patients with samples from both time points in Cohort A.

The patient demographic and injury-related details are shown in Table 1, and are compared between the two cohorts. Patients in Cohort B had worse physiological parameters for haemodynamic shock and coagulopathy when compared to Cohort A, with lower median SBP (90 *vs* 114; *p* = 0.0002) and HR (105 vs 87; *p* = 0.0004), as well as higher INR and PTT ratio (both *p* = 0.020). Otherwise their demographic and injury patterns were not significantly different between cohorts.

**Table 1:** Patient characteristics according to patient cohort.

Characteristic	All (N = 122)	Cohort A (N = 105)	Cohort B (N = 17)	*p*-value
**Age, mean (SD)**	41 (19)	41 (20)	40 (18)	0.816
**Male, N (%)**	108 (87)	90 (86)	16 (94)	0.466
**BMI, median (IQR)**	25 (23–28)	25 (23–28)	25 (22–29)	0.891
**Smoker, N (%)**	55 (45)	47 (45)	8 (47)	>0.999
**Alcohol intoxication, N (%)**	19 (16)	19 (18)	0 (0)	0.071
**Co-morbidities**				
N (%)	26 (21)	23 (22)	3 (18)	>0.999
CCI, median (range)	0 (0–5)	0 (0–5)	0 (0–4)	0.814
**ISS, median (IQR)**	25 (12–34)	24 (10–36)	27 (17–34)	0.446
**Injury mechanism, N (%)**				
Blunt	101 (81)	86 (82)	13 (76)	0.738
Penetrating	23 (19)	19 (18)	4 (24)	
**GCS, median (IQR)**	12 (4–15)	13 (4–15)	9 (3–14)	0.232
**Lactate (mmol/l), median (IQR)**	3.7 (2.5–6.4)	3.5 (2.5–6.3)	6.0 (3.6–10.2)	0.071
**SBP (mmHg), median (IQR)**	111 (97–122)	114 (99–124)	91 (61–108)	0.0002^a^
**HR (min**^**-1**^**), median (IQR)**	89 (79–100)	87 (76–99)	108 (98–118)	0.0004^a^
**INR, median (IQR)**	1.1 (1.0–1.2)	1.1 (1.0–1.2)	1.2 (1.1–1.2)	0.020^a^
**PTT ratio, median (IQR)**	0.9 (0.8–1.0)	0.9 (0.8–0.9)	0.9 (0.9–1.1)	0.020^a^
**Outcomes**				
Hospital-free days, median (IQR)	6 (0–20)	8 (0–20)	0 (0–5)	0.097
ICU-free days, median (IQR)	20 (8–29)	22 (10–29)	8 (0–18)	0.003^a^
Mortality, n (%)	19 (16)	16 (15)	3 (18)	0.728
PE, n (%)	1 (0.8)	1 (1.0)	0 (0)	>0.999

All continuous data are presented as either mean or median, with standard deviation or interquartile range in parentheses respectively, as indicated (with the exception that Charlson Comorbidity Index is shown with range in parentheses); categorical data are presented as N, with percentage in parentheses.

^a^Statistically significant according to Mann-Whitney test

BMI: body mass index; CCI: Charlson Comorbidity Index; ISS: injury severity score; GCS: Glasgow Coma Scale; SBP: systolic blood pressure; HR: heart rate; INR: international normalised ratio; PTT: partial thromboplastin time; ICU: Intensive Care Unit; PE: pulmonary embolism

Cohort B had a significantly lower number of ICU-free days than those in Cohort A (8 *vs* 22; *p* = 0.003), but there were no significant differences in other outcomes between groups.

When patients who received blood product transfusion were compared to those with no transfusion, they had a higher median lactate (6.2 mmol/l vs 3.2 mmol/l; p<0.0001), lower median SBP (93 mmHg vs 116 mmHg; p<0.0001), and higher median HR (97 min^-1^ vs 87 min^-1^; *p* = 0.018) (Table 2). Although the transfused group had a higher median ISS than the non-transfused group, this did not reach statistical significance.

**Table 2:** Patient characteristics according to requirement for transfusion.

Characteristic	All (N = 122)	Received transfusion (N = 31)	No transfusion (N = 91)	*p*-value
**Age, mean (SD)**	41 (19)	43 (21)	41 (19)	0.503
**Male, n (%)**	108 (87)	27 (87)	79 (87)	>0.999
**ISS, median (IQR)**	25 (12–34)	27 (16–43)	23 (10–30)	0.061
**Injury mechanism, n (%)**				
Blunt	101 (81)	23 (74)	76 (84)	0.290
Penetrating	23 (19)	8 (26)	15 (16)	
**GCS, median (IQR)**	12 (4–15)	10 (3–14)	13 (4–15)	0.133
**Lactate (mmol/l), median (IQR)**	3.7 (2.5–6.4)	6.2 (4.0–9.5)	3.3 (2.3–5.1)	<0.0001^a^
**SBP (mmHg), median (IQR)**	111 (97–122)	93 (69–109)	116 (103–124)	<0.0001^a^
**HR (min**^**-1**^**), median (IQR)**	89 (79–100)	97 (81–115)	87 (76–99)	0.018^a^

All continuous data are presented as mean or median, with standard deviation or interquartile range in parentheses respectively; categorical data are presented as N, with percentage in parentheses.

^a^Statistically significant according to Mann-Whitney test

ISS: injury severity score; GCS: Glasgow Coma Scale; SBP: systolic blood pressure; HR: heart rate

### Association between endothelial biomarkers and cfDNA

**Fig 2:**
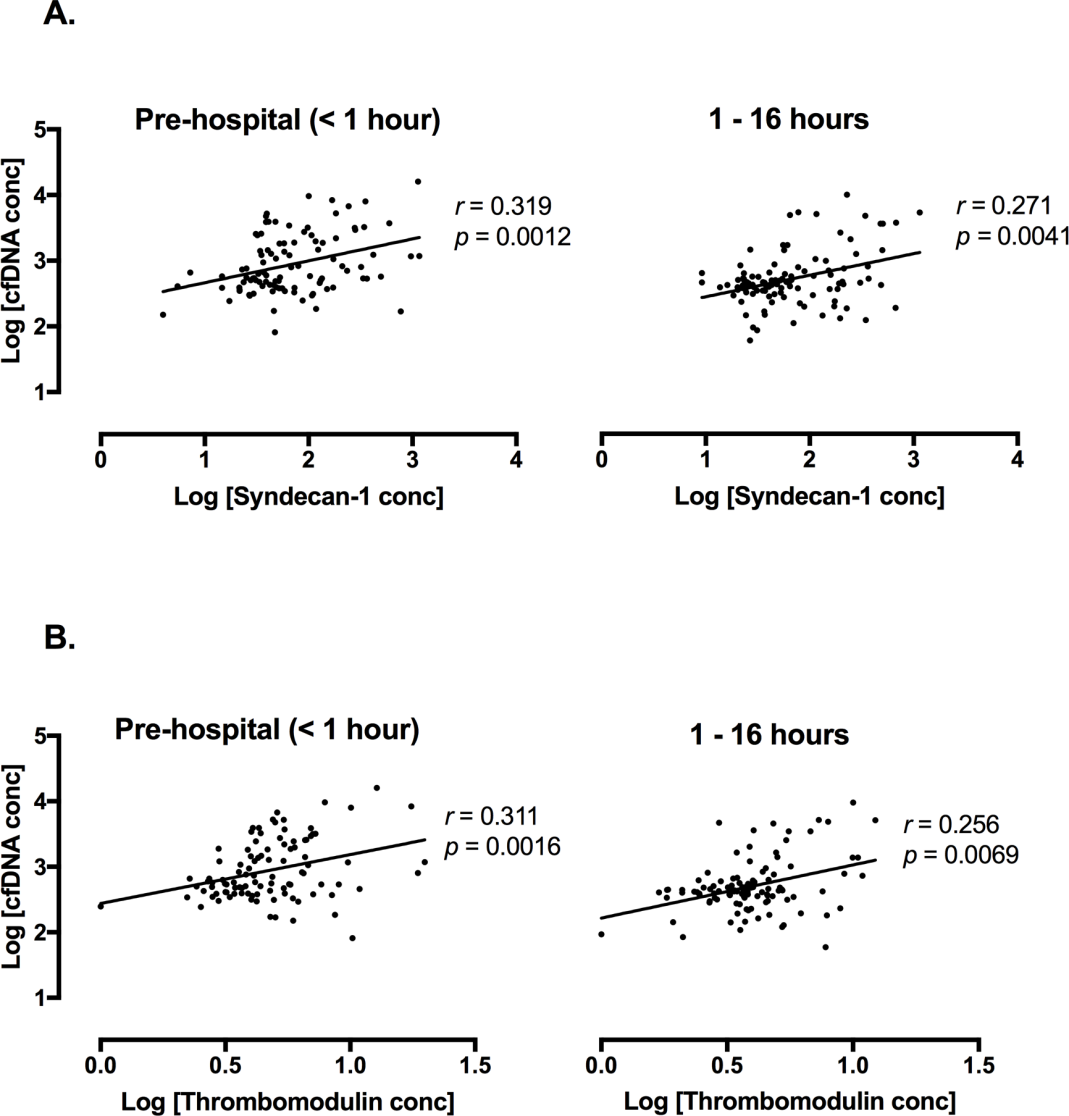
**Association between (a) syndecan-1 and (b) thrombomodulin and cell-free DNA (cfDNA) according to time points.** *P*-values are indicated according to Spearman’s rank correlation coefficient. For both concentrations of (a) syndecan-1 and (b) thrombomodulin, there is a significant correlation with concentration of cfDNA at both time points.

**Fig 3:**
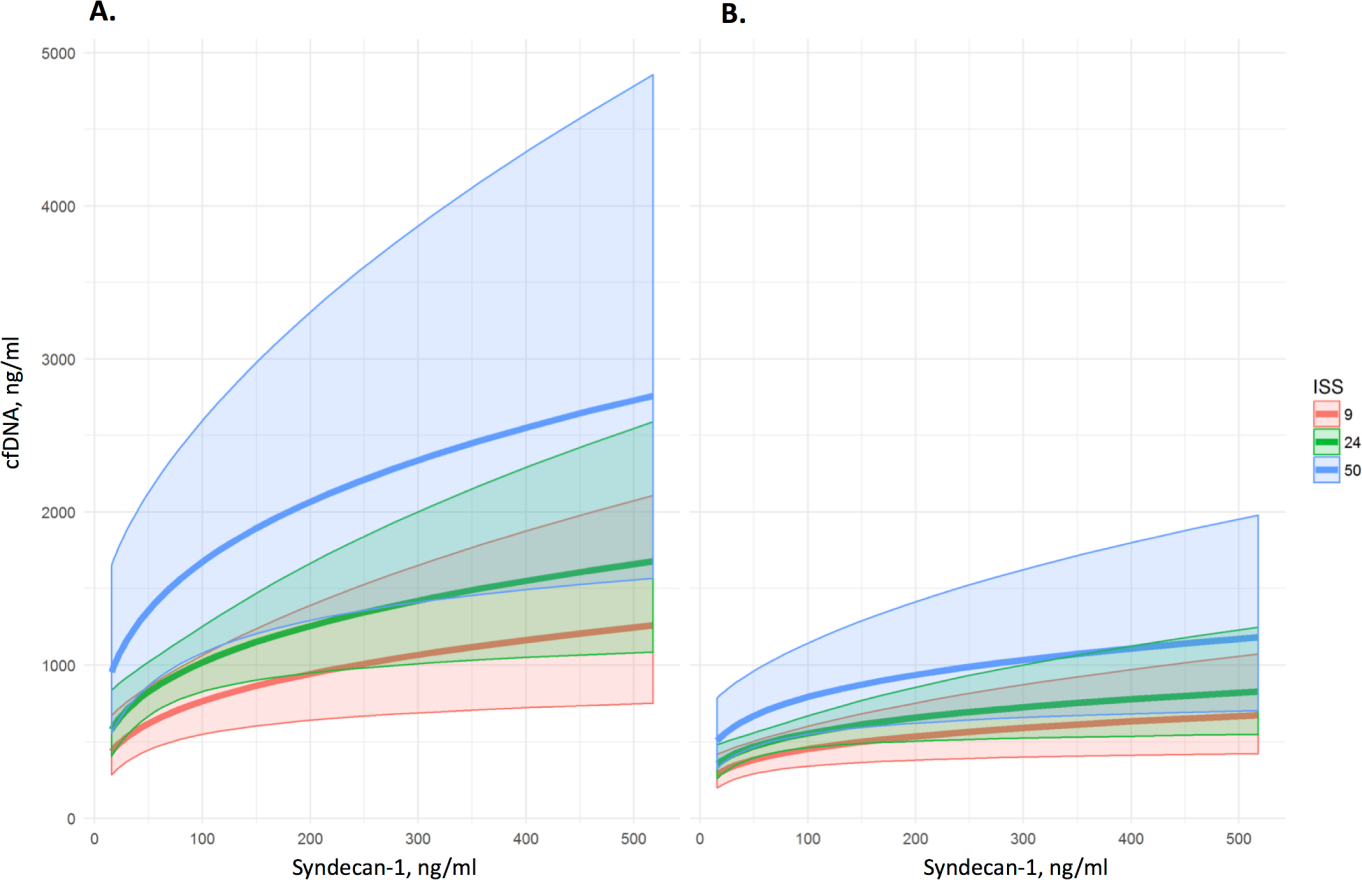
**Relationship between cell-free DNA and syndecan-1 at (a) first (prehospital) time point and (b) second (in-hospital) time point, according to category of increasing injury severity.** Solid lines represent the mean predicted values (based on the fitted model), and the shaded areas represent the range of values covered by the 95% confidence intervals associated with those predicted values.

When samples were analysed at each specific time point (N = 100 for pre-hospital, and N = 110 for 1–16h time points), there were significant correlations between cfDNA and both syndecan-1 and thrombomodulin at both time points (Fig 2). When Cohorts A and B were individually analysed, there were similarly significant correlations between cfDNA concentration and concentrations of syndecan-1 and thrombomodulin (all *p*<0.05). Using multivariable linear regression, the relationships between cfDNA and both syndecan-1 and thrombomodulin were independent of gender, age, GCS, lactate, SBP, and HR. However, there was an additional significant relationship between ISS and cfDNA. The modelled relationship between cfDNA and syndecan-1 at increasing ISS values can be visualised in Fig 3.

### Blood transfusion and surgery

When patients were divided into those who had surgery in between time points and those that did not, there were 43/122 (35%) patients who had surgery. There were no significant differences in cfDNA, syndecan-1, or thrombomodulin levels at either baseline or at the second time point between those who had surgery between time points and those who did not (Table 3).

**Table 3:** Biomarker concentrations at both time points compared between patients that had surgery between time points and those who did not.

Biomarker	Had surgery (N = 43)	No surgery (N = 79)	*p*-value
**Prehospital biomarkers, ng/ml**			
cfDNA	558 (384–956)	817 (422–2442)	0.132
Syndecan-1	49 (35–118)	59 (33–136)	0.700
Thrombomodulin	4.3 (3.4–6.2)	4.7 (3.7–6.3)	0.508
**1-16h biomarkers, ng/ml**			
cfDNA	411 (233–1141)	457 (368–609)	0.231
Syndecan-1	65 (37–197)	46 (26–111)	0.110
Thrombomodulin	4.0 (3.5–5.4)	3.7 (3.0–4.6)	0.087

All summary data are presented as median, with interquartile range in parentheses.

cfDNA: cell-free DNA

When patients were divided into those who had blood transfusion in between time points and those who did not, there were 31/122 (25%) who were transfused. There were no significant differences in cfDNA, syndecan-1, or thrombomodulin levels between groups at baseline (Table 4). After blood product transfusion in ED, there were no differences in cfDNA or thrombomodulin between the transfused and non-transfused groups, but there was a significantly higher concentration of syndecan-1 amongst the transfused group (107 ng/ml vs 43 ng/ml; *p* = 0.0005) (Table 4).

**Table 4:** Biomarker concentrations at both time points compared between patients that received a blood transfusion and those who did not.

Biomarker	Received transfusion (N = 31)	No transfusion (N = 91)	*p*-value
**Prehospital biomarkers, ng/ml**			
cfDNA	828 (379–3174)	645 (410–1839)	0.501
Syndecan-1	109 (35–281)	50 (34–118)	0.095
Thrombomodulin	5.7 (3.5–7.1)	4.4 (3.6–5.9)	0.248
**1-16h biomarkers, ng/ml**			
cfDNA	466 (299–1790)	433 (346–570)	0.191
Syndecan-1	107 (43–256)	43 (25–89)	0.0005^a^
Thrombomodulin	4.1 (3.5–5.2)	3.7 (2.9–4.7)	0.073

All summary data are presented as median, with interquartile range in parentheses.

^a^significant according to Mann-Whitney test

cfDNA: cell-free DNA

### Biomarkers and coagulopathy

Table 5 shows the correlations between the three biomarkers (cfDNA, syndecan-1, and thrombomodulin) at both time points, and both admission INR and PTT ratios. Syndecan-1 levels were significantly correlated with INR and PTT ratios at both the pre-hospital (*p* = 0.036 and *p* = 0.002 respectively) and in-hospital (*p* = 0.010 and *p*<0.0001 respectively) time points. cfDNA levels were only significantly correlated with INR at the prehospital time point (with a borderline significant *p-*value of 0.048), and with PTT ratio at the in-hospital time point (*p* = 0.028). Thrombomodulin was only significantly correlated with PTT ratio at the in-hospital time point (*p* = 0.001).

**Table 5:** Correlations between biomarker concentrations at both pre-hospital (<1h) and in-hospital (1-16h) time points and admission INR and PTT ratios.

Biomarker	Correlation with biomarkers, *r* (95% CI)			
	INR	*p*-value	PTT ratio	*p*-value
**Prehospital biomarkers**				
cfDNA	0.195 (0.00–0.380)	0.048^a^	0.000 (-0.218–0.220)	0.993
Syndecan-1	0.208 (0.01–0.392)	0.036^a^	0.338 (0.125–0.521)	0.002^a^
Thrombomodulin	0.119 (-0.084–0.311)	0.119	0.195 (-0.029–0.399)	0.078
**1-16h biomarkers**				
cfDNA	0.054 (-0.157–0.259)	0.607	0.222 (0.018–0.407)	0.028^a^
Syndecan-1	0.243 (0.053–0.415)	0.010^a^	0.428 (0.244–0.582)	<0.0001^a^
Thrombomodulin	0.085 (-0.109–0.272)	0.377	0.328 (0.132–0.500)	0.001^a^

All summary data are presented as *r*, with 95% confidence intervals in parentheses.

^a^Significant according to Spearman’s rank correlation coefficient

INR: international normalised ratio; PTT: partial thromboplastin time; cfDNA: cell-free DNA

### Biomarker levels over time

**Fig 4:**
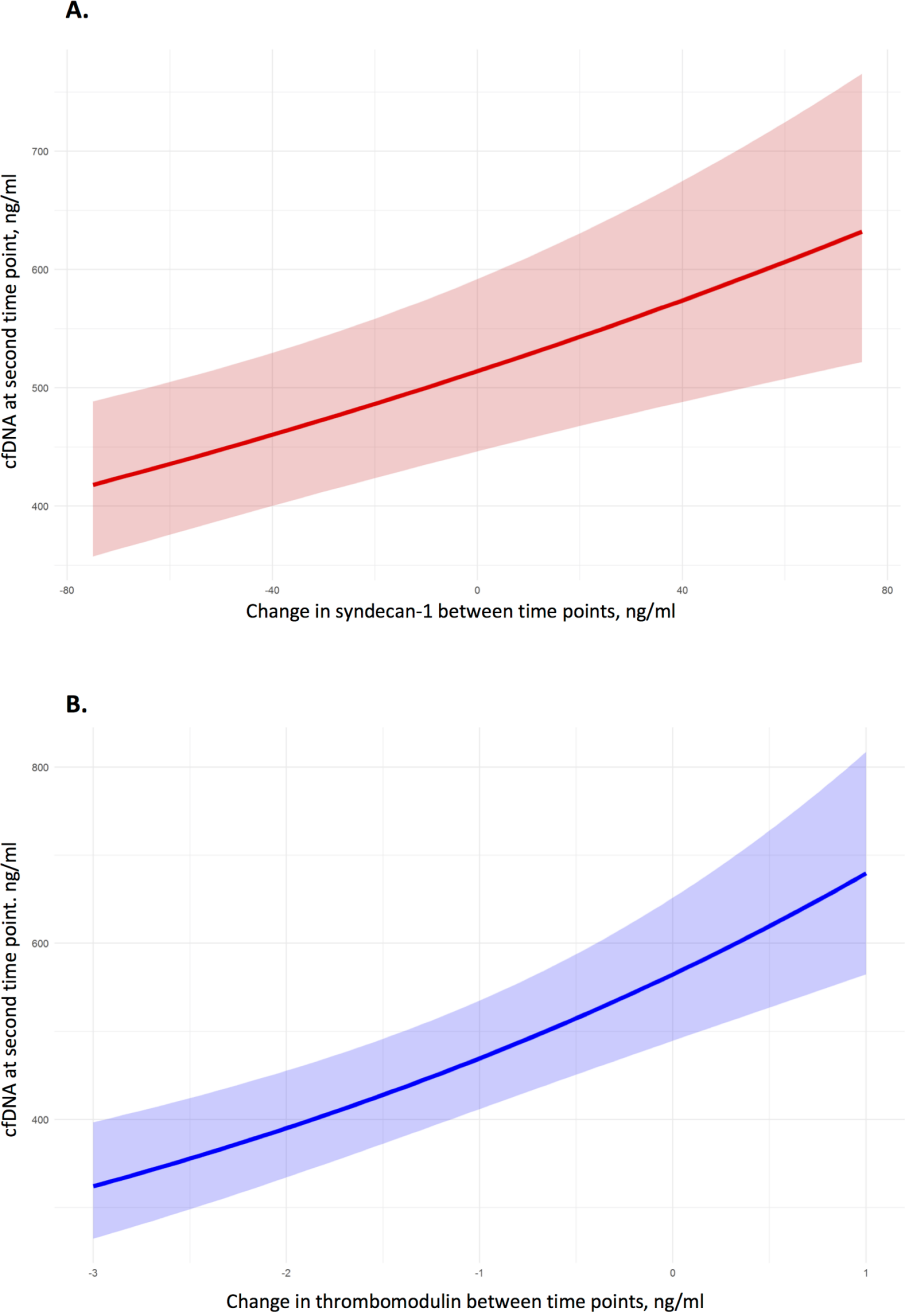
**Relationship between cfDNA levels at the second time point and change in (a) syndecan-1 and (b) thrombomodulin concentrations between time points.** Solid lines represent the mean predicted values (based on the fitted model), and the shaded areas represent the range of values covered by the 95% confidence intervals associated with those predicted values.

For patients in Cohort A with cfDNA results for both time points (n = 88), the differences in cfDNA concentrations between time points were compared to differences in concentrations of biomarkers of endotheliopathy. When a model was fitted to examine the value of cfDNA at the second time point as a function of the change in syndecan-1 and thrombomodulin levels, increases in the change in syndecan-1 and thrombomodulin between time points were associated with statistically significant increases in cfDNA levels, even accounting for the cfDNA value at the first time point. With all other covariates held at fixed values, a 50 ng/ml change in syndecan-1 between time points corresponded to a 15% increase in cfDNA levels (95% CI 7–23%; *p* = 0.0002) (Fig 4A), and a 1 ng/ml change in thrombomodulin between time points corresponded to a 20% increase in cfDNA levels (95% CI 12–29%; *p*<0.0001) (Fig 4B).

### Biomarker levels and outcomes

**Fig 5:**
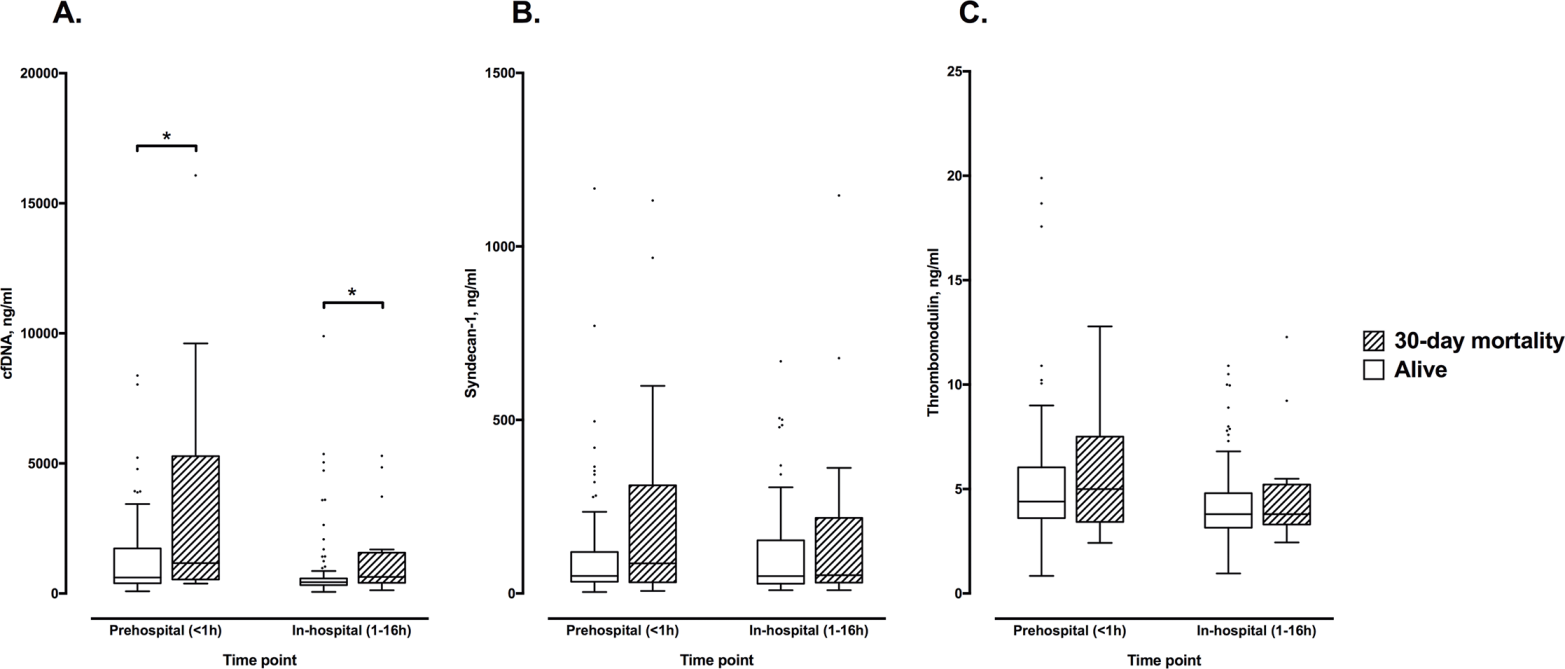
**Comparison at both time points according to 30-day mortality for levels of (a) cell-free DNA; (b) syndecan-1; and (c) thrombomodulin.** *p<0.05 according to Mann-Whitney *U* test.

When cfDNA levels were compared between those who died within 30 days and those who survived, those who died had significantly higher levels at both the prehospital (1170 (IQR 538–5279) ng/ml *vs* 617 (IQR 391–1731) ng/ml; borderline *p-*value of 0.049) and in-hospital (636 (IQR 410–1569) ng/ml *vs* 432 (IQR 322–577) ng/ml; *p* = 0.030) time points (Fig 5A). There were no significant differences between groups in terms of survival for the prehospital or in-hospital levels of syndecan-1 (Fig 5B) or thrombomodulin (Fig 5C).

When hospital-free days and ICU-free days were compared to both prehospital and in-hospital biomarkers, there were significant negative correlations between cfDNA and syndecan-1 levels and these outcomes, but thrombomodulin levels did not have any significant correlations (Table 6). Only one patient had a thromboembolic event (Table 1), therefore no comparisons could be made with biomarker levels.

**Table 6:** Correlations between biomarker concentrations at both pre-hospital (<1h) and in-hospital (1-16h) time points and length of stay outcomes.

Biomarker	Correlation with outcomes, *r* (95% CI)			
	Hospital-free days	*p*-value	ICU-free days	*p*-value
**Prehospital biomarkers**				
cfDNA	-0.390 (-0.546 –-0.207)	<0.0001^a^	-0.385 (-0.542 –-0.201)	<0.0001^a^
Syndecan-1	-0.217 (-0.399 –-0.017)	0.029^a^	-0.187 (-0.373–0.014)	0.060
Thrombomodulin	-0.140 (-0.331–0.062)	0.161	-0.130 (-0.321–0.072)	0.194
**1-16h biomarkers**				
cfDNA	-0.331 (-0.490 –-0.150)	0.0003^a^	-0.334 (-0.493 –-0.154)	0.0003^a^
Syndecan-1	-0.224 (-0.399 –-0.034)	0.018^a^	-0.207 (-0.384 –-0.016)	0.029^a^
Thrombomodulin	-0.165 (-0.346–0.028)	0.083	-0.178 (-0.357–0.015)	0.062

All summary data are presented as *r*, with 95% confidence intervals in parentheses.

^a^Significant according to Spearman’s rank correlation coefficient

ICU: intensive care unit; cfDNA: cell-free DNA

## Discussion

The main finding from the current study is that there is a strong association between circulating levels of cfDNA and biomarkers of endotheliopathy following trauma, independent of physiological parameters. Neither transfusion nor surgery were associated with higher levels of cfDNA. There were strong associations between levels of cfDNA and clinical outcomes. These associations were observed in both pre-hospital (<1h) and in-hospital (1-16h) environments, and changes in endothelial biomarkers had the same directional change as those of cfDNA between these time points. These findings suggest that the presence of cfDNA within the circulation and endotheliopathy of trauma may be mechanistically linked, confirming findings of previous preclinical [10, 23, 28, 29] and clinical [15, 24, 25] studies. To our knowledge, our study is the first to report this association within the pre-hospital period of evacuation following injury, supporting the hypothesis that this is an early phenomenon, occurring within minutes, rather than hours, of injury. The half-life of cfDNA has been reported as under 2 hours [30], which implies that persistently elevated levels of cfDNA over subsequent days may be due to sustained production or reduced clearance by DNAse. Since the half-lives are similarly short for syndecan-1 [31, 32] and thrombomodulin [33], our findings that the rise or fall in cfDNA is associated with similar patterns in endothelial biomarker levels suggest that they may share a common pathway.

An observational clinical study of this kind cannot demonstrate a causal link between endotheliopathy and increase in release of cfDNA, or whether an increase in cfDNA from injured tissues causes endotheliopathy, making preclinical data the best source for hypotheses. There is evidence that NETs cause damage to endothelial cells due to the degradation of VE-cadherin and activation of β-catenin signalling [29]. When NETs are free in the circulation, they may promote tissue injury and oedema [34], and can bind to endothelial cells and cause direct injury [28]. After prolonged exposure to NETs, endothelial cells are more likely to die than non-exposed cells or those only exposed for a short period of time [35]. We propose that the most likely sequence of events is that cfDNA from cellular injury, surgical intervention, and the production of NETs by circulating activated immune cells injures the vascular endothelium, which in turn releases new cfDNA.

There has been recent evidence that in addition to endogenous sources of cfDNA, injured patients may be exposed to further fragments of mtDNA from transfused packed red blood cells, plasma, and platelets, and that delivery of these to patients may increase the risk of transfusion-related lung injury and acute respiratory distress syndrome [36, 37]. In the current study, blood transfusion was not associated with higher levels of cfDNA, even when the transfusion group had worse parameters for perfusion (lactate) and haemodynamic compromise (SBP and HR). The significantly higher syndecan-1 levels in the transfused group may represent a greater amount of glycocalyx shedding amongst the patients with haemorrhagic shock, a finding in keeping with other studies of the endothelium following trauma[38]. Although we were not able to confirm the previous findings that transfusion may increase the amount of cfDNA, these were a heterogeneous group of patients, and non-randomised. It is unknown whether differences may have occurred after our study time points (i.e. >16 hours).

Biomarkers such as cfDNA, syndecan-1, and thrombomodulin have not yet entered clinical utility, and their roles within diagnosis and management of trauma are still uncertain. Since the current study has shown that raised levels of these biomarkers are associated with poorer clinical outcomes such as mortality and length of stay in hospital or ICU, they may therefore have some prognostic value. Their potential role in diagnosis and treatment is not yet known. Best practice guidelines recommend the use of goal-directed therapy aimed at correcting coagulopathy using viscoelastic assays such as rotational thromboelastometry or thromboelastography [39]. Since cfDNA, thrombomodulin, and syndecan-1 have all been shown to be associated with coagulopathy[8, 9, 14, 15, 17, 18], a finding that was confirmed in the current study, they represent possible sources of further information (in addition to viscoelastic assays) to the trauma clinician during resuscitation. A previous investigation reported better patient outcomes when there was a decrease in cfDNA between time points [40]. In our study, a decrease in cfDNA was associated with a similar decrease in both syndecan-1 and thrombomodulin. The reduction in these biomarkers suggests a relative restoration of the endothelium and reduction in cell injury and NET production, which may partly explain any better clinical outcomes previously observed.

The current study combines analysis of blood samples from two different cohorts of trauma patients; Cohort A included patients who had any form of injury as long as it was likely to have an ISS >8, whereas Cohort B included patients with severe injuries and the additional burden of haemorrhagic shock. Despite the differences in these cohorts, there was an equally compelling relationship between cfDNA and endotheliopathy within each cohort as well as within the whole group. Furthermore, the association between syndecan-1, thrombomodulin, and cfDNA remained consistent independently of physiological and transfusion status. It is tempting to conclude that these biomarkers represent a common pathway. However, it is possible that other confounding variables are responsible for the elevation and decrease in both cfDNA and endothelial biomarkers. For example, levels of mtDNA have been linked to surgical trauma, injury severity and volume of intravenous fluids delivered [41, 42]. The current study shows a relationship between ISS and cfDNA. The precise relationship between cfDNA and the endothelium, although examined in detail in preclinical studies, requires further analysis in humans. In particular, attention should be given to causality between these biomarkers, so that a full narrative of trauma from initial injury to endotheliopathy, coagulopathy, and organ dysfunction might be better understood.

### Limitations

The current study reports a relatively high burden of injury (median ISS of 25) amongst patients, and therefore the findings may not necessarily be translatable to less severely injured patients. However, the range in ISS and injury mechanisms within the study cohorts may increase the reliability and translatability of the main findings. The number of patients is relatively low, and from a single Major Trauma Centre in a developed trauma network. Our findings may benefit from corroboration in further sites and other trauma systems. In particular, our trauma network does not currently deliver pre-hospital blood products, and it is unknown what effects these may have on cfDNA and endothelial biomarkers before arrival in hospital.

Although we report markers of endotheliopathy, the clinical impact (such as the potential for soft tissue oedema and alterations to normal fluid balance) was not examined in the current study. Thromboelastography was not available during this study. Instead, INR was used as a marker of acute traumatic coagulopathy as previously described[43]. Further clinical investigations of the impact of endotheliopathy on vascular permeability and thromboelastography are warranted.

### Conclusions

We report an association between concentrations of cfDNA and markers of endotheliopathy (syndecan-1 and thrombomodulin) within the circulation following injury that is independent of physiological parameters. This relationship is present within the first hour of injury, persists over time, with an increase or decrease in concentration of one biomarker being reflected by a similar change in the others. There is an association between cfDNA and injury severity, and higher levels of cfDNA are associated with longer lengths of stay and mortality. Although causality cannot be established, these findings are in keeping with previous evidence that there is a close mechanistic relationship between circulating DNA, vascular endothelial injury, and poorer clinical outcomes.

## Supporting information

S1 File Database of the study's raw data. (XLSX)
